# The Influence of 0.5% Tropicamide on Anterior Segment Parameters With CASIA2 in Emmetropic, Myopic, and Hyperopic Eyes

**DOI:** 10.3389/fphys.2022.957097

**Published:** 2022-07-12

**Authors:** Feng Lin, Yuliang Wang, Yujia Liu, Xiaomei Qu, Xingtao Zhou

**Affiliations:** ^1^ Eye Institute and Department of Ophthalmology, Eye & ENT Hospital, Fudan University, Shanghai, China; ^2^ NHC Key Laboratory of Myopia (Fudan University); Key Laboratory of Myopia, Chinese Academy of Medical Sciences, Shanghai, China; ^3^ Shanghai Research Center of Ophthalmology and Optometry, Shanghai, China; ^4^ Shanghai Engineering Research Center of Laser and Autostereoscopic 3D for Vision Care, Shanghai, China

**Keywords:** tropicamide, anterior segment parameters, casia2, emmetropia, myopia, hyperopia

## Abstract

**Aim:** To evaluate the effects of 0.5% tropicamide on anterior segment parameters with the CASIA2 imaging device in emmetropic, myopic, and hyperopic eyes.

**Methods:** In this prospective study, a total of 125 subjects (34 emmetropic subjects, 57 myopic subjects, and 34 hyperopic subjects) at the Shanghai Eye and ENT Hospital of Fudan University were recruited from June 2021 to September 2021. The 0.5% tropicamide solution was used once every 5 min a total of 5 times for cycloplegia. The anterior segment parameters were recorded by CASIA2 before and after cycloplegia. Changes in anterior segment parameters were compared among the three refractive groups.

**Results:** Crystalline lens rise (CLR) and crystalline lens thickness (CLT) significantly decreased in all three refractive groups after cycloplegia (all *p* < 0.01). The anterior radius of lens (ARL) and anterior chamber depth (ACD) significantly increased in all three refractive groups after cycloplegia (all *p* < 0.01). Posterior radius of lens (PRL) significantly increased in hyperopic eyes after cycloplegia (*p* < 0.01) while it remained unchanged in emmetropic eyes and myopic eyes. Central corneal thickness (CCT), anterior chamber width (ACW), lens decentration (LD), and lens tilt (LT) remained unchanged after cycloplegia in all three refractive groups (all *p* > 0.05). Changes in CLR, CLT, ARL, PRL, and ACD in hyperopic eyes were greater than those in emmetropic eyes and myopic eyes (all *p* < 0.05).

**Conclusion:** Apart from various changes in anterior segment parameters after application by 0.5% tropicamide in all three refractive groups, significant changes in CLR, CLT, ARL, PRL, and ACD in hyperopic eyes should be noted for proper clinical interpretation.

## Introduction

As children have strong accommodation abilities, cycloplegic refraction is very important. (Lin et al., 2017) With cycloplegic drugs, pseudomyopia and latent hyperopia can be identified. Commonly used mydriatics include tropicamide, cyclopentolate, atropine, and phenylephrine. (Yazdani et al., 2018) Among these, tropicamide has become popular in China, as it causes rapid onset of cycloplegia. However, while mydriatics reduce ciliary muscle spasm, they may also have an effect on the anterior segment parameters. (Palamar et al., 2011; Chen et al., 2020; Dai et al., 2021) Cyclopentolate hydrochloride reportedly leads to a significant increase in anterior chamber depth (ACD) and anterior chamber volume (ACV), and significantly decreases central corneal thickness (CCT). (Palamar et al., 2011) In addition, anterior segment parameters are quite important in determining the suitability for phakic intraocular lens (pIOL) and implantable contact lens (ICL) surgery and evaluating various diseases such as glaucoma and keratoconus. (Yan et al., 2010; Nongpiur et al., 2011; Gonzalez-Lopez et al., 2019; Yağmur Kanra and Uslu, 2021) Therefore, for proper clinical interpretation it is important to consider the effect of mydriatics on anterior segment parameters.

CASIA2 imaging device (Tomey Corporation, Nagoya, Japan), the second generation of anterior segment optical coherence tomography (AS-OCT), has good repeatability and reproducibility. (Shoji et al., 2017; Fukuda et al., 2020) The results obtained by CASIA2 were not influenced by pupil dilation and showed good repeatability under both non-mydriatic and mydriatic conditions. (Kimura et al., 2017) In addition, CASIA2 has a scanning speed of 50 ,000 A-scans per second and a scanning depth of 13 mm. (Shoji et al., 2017) By thus, CASIA2 can acquire classic anterior chamber parameters such as CCT and ACD as well as detailed lens parameters (such as crystalline lens rise [CLR], anterior radius of lens [ARL], and posterior radius of lens [PRL]). With CASIA2, a more comprehensive understanding of the effects of mydriatics on the anterior segment parameters is possible. While tropicamide has been widely used in clinical practice, tropicamide reportedly decreases CLR and increases ACD in myopic eyes with CASIA2. (Chen et al., 2020) However, as tropicamide causes different cyclopegic effects depending on each refractive status, its effects on anterior segment parameters may vary in emmetropic, hyperopic, and myopic eyes. (Yazdani et al., 2018) The effect of tropicamide on anterior segment parameters with CASIA2 in hyperopic eyes remains unknown. In addition, whether the effects of tropicamide on the anterior segment parameters in the three refractive statuses are consistent with each other is also worthy of further analysis.

In this study, we reported changes in anterior segment parameters after cycloplegia caused by 0.5% tropicamide while using CASIA2 on emmetropic, myopic, and hyperopic eyes. In addition, changes in these parameters were compared among the three refractive statuses to provide more detailed reference data for proper clinical interpretation of anterior segment parameters after administration of tropicamide.

## Materials and Methods

### Participants

In this prospective study, 130 subjects who visited the Shanghai Eye and ENT Hospital of Fudan University from June 2021 to September 2021 were recruited. The inclusion criteria for the subjects were age between 4 and 15 years and astigmatism less than 2.0D. The exclusion criteria were acute ocular surface inflammation, a history of ocular surgery, a history of ocular trauma, a history of administration of atropine and a history of wearing orthokeratology (OK) lens. This study, approved by the Ethics Committee of the Shanghai Eye and ENT Hospital, adhered to the tenets of the Declaration of Helsinki. Written informed consent was obtained from the parents of the subjects.

### Anterior Segment Measurements

CASIA2 is a second generation of AS-OCT at a wavelength of 1310 nm. It achieves fast scanning speed 50 ,000 A-scans per second, high resolution imaging (10 μM axially and 30 μM transversally), and deep scanning depth (13 mm). The measurement range of CASIA2 is φ16 mm × 13 mm. With Fourier Domain method, CASIA2 allows clear three-dimensional (3D) imaging of the anterior segment. The CASIA2 takes 0.016 s to capture a single cross-sectional image, producing 128 cross-sectional images evenly spaced 1.4° apart in each scan session.

**FIGURE 1:**
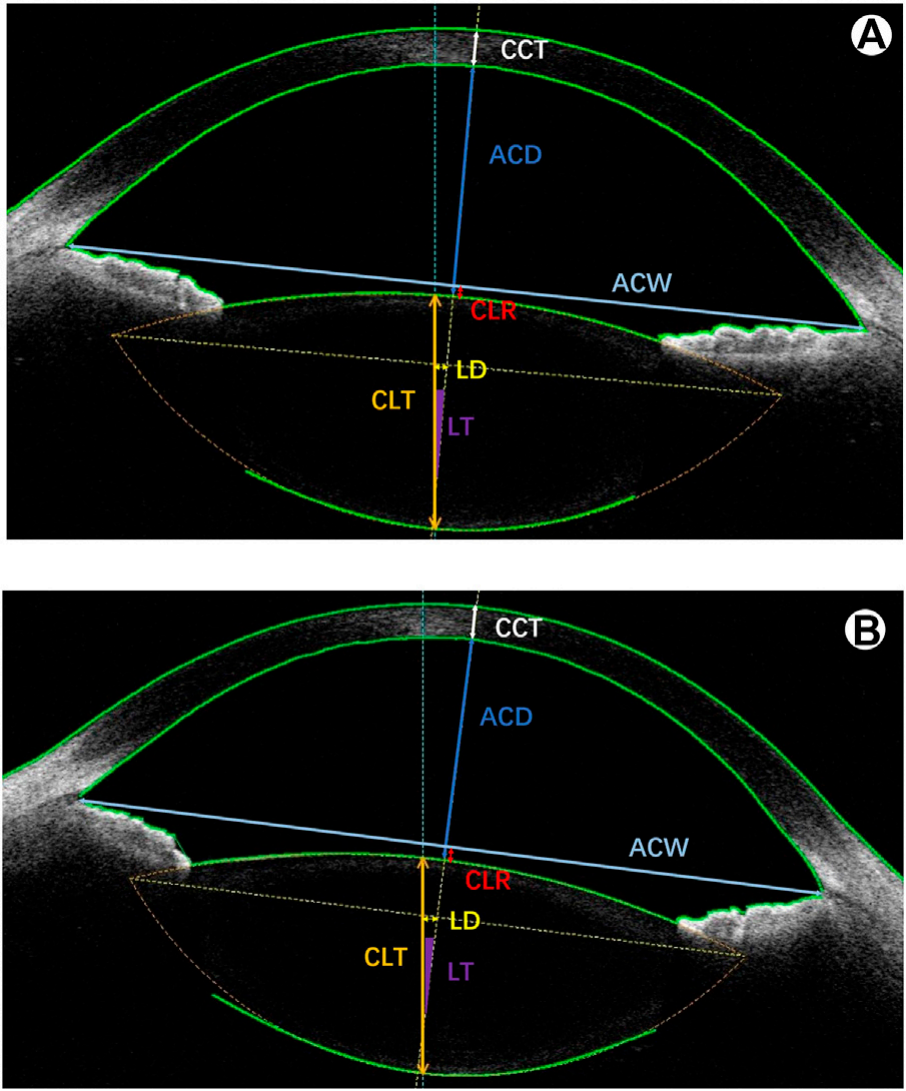
A representative image with anterior segment parameters of the CASIA2. **(A)** before cycloplegia; **(B)** after cycloplegia. CCT: central corneal thickness; ACD: anterior chamber depth; ACW: anterior chamber width; CLR: crystalline lens rise; CLT: crystalline lens thickness; LD: lens decentration; LT: lens tilt.

Before cycloplegia, anterior segment parameters were obtained using CASIA2. These parameters included CCT, CLR, crystalline lens thickness (CLT), ARL, PRL, ACD, anterior chamber width (ACW), lens decentration (LD) and lens tilt (LT) (Figure 1). Then, 0.5% tropicamide eye drops were administered to both eyes of a subject once every 5 min a total of 5 times. The examinations of the anterior segment parameters with CASIA2 were again carried out 30 min later. Cycloplegic retinoscopy was then performed by an experienced optometrist.

Each of the measurements with CASIA2 was conducted three times for accuracy. In addition, parameters of both eyes were recorded, and only the right eye of each subject was selected for analysis in this study.

### Definition of Refractive Groups

Subjects were divided into an emmetropic group, myopic group, and hyperopic group according to the spherical equivalent (SE) of their eyes after cycloplegia. SE was calculated based on a formula (SE = spherical + astigmatism/2). Emmetropia was defined as SE between -0.75D and +1.0D. Myopia was defined as SE equal to or less than −0.75D. (Zadnik et al., 2015) Hyperopia was defined as SE equal to or greater than +1.0D. (Lee et al., 2004)

### Statistical Analysis

The results are represented as mean ± standard deviation. All parameters were tested using the Kolmogorov–Smirnov test for normality of the data distribution. Anterior segment parameters before and after cycloplegia were compared by paired *t*-test (normally distributed parameters) or Wilcoxon signed-rank test (non-normally distributed parameters). Differences in sex, age, and changes in anterior segment parameters among the three refractive groups were compared by one-way analysis of variance (normally distributed parameters), Kruskal–Wallis test (non-normally distributed parameters), or Chi-squared test (categorical variables). *p <* 0.05 was considered statistically significant. SPSS version 25.0 software (IBM, Chicago, IL) was adopted to perform the analysis.

## Results

### Baseline Characteristics

A total of 125 eyes from 125 subjects (64 males and 61 females) were included in the study. The mean age of the subjects was 8.37 ± 2.11 years (range: 4–13 years). All eyes were divided into three refractive groups that comprised 34 emmetropic eyes, 57 myopic eyes, and 34 hyperopic eyes. The emmetropic group had a refractive SE status range of −0.625D to +0.875D. The myopic group had a refractive SE status range of −6.375D to −0.75D. The hyperopic group had a refractive SE status range of +1.0D to +6.875D.

Table 1 shows the subject demographics of the three refractive groups. No significant difference was found in the age and sex among the three refractive groups (all *p* > 0.05).

**TABLE 1:** Subject demographics of emmetropic, myopic and hyperopic eyes.

	Emmetropia (*n* = 34)	Myopia (*n* = 57)	Hyperopia (*n* = 34)	*p* value
SE (D)	0.00 ± 0.43	−2.72 ± 1.58	3.69 ± 1.75	<0.01^a^
Age	8.35 ± 2.31	8.47 ± 1.93	8.21 ± 2.25	0.839^a^
Sex (male:female)	18:16	30:27	16:18	0.852^b^

a Kruskal-Wallis test.

b Chi-square test.

SE, spherical equivalent.

### Anterior Segment Parameters Before and After Cycloplegia in Emmetropic, Myopic, and Hyperopic Eyes

Table 2 shows the anterior segment parameters before and after cycloplegia in emmetropic, myopic, and hyperopic eyes. CLR significantly decreased from 3.26 ± 143.39 µM to −35.53 ± 152.72 µM in emmetropic eyes, from −66.36 ± 151.97 µM to −95.75 ± 143.70 µM in myopic eyes, and from 176.97 ± 160.64 µM to 32.65 ± 136.28 µM in hyperopic eyes (all *p* < 0.01). CLT significantly decreased from 3.42 ± 0.18 mm to 3.38 ± 0.19 mm in emmetropic eyes, from 3.37 ± 0.16 mm to 3.34 ± 0.15 mm in myopic eyes, and from 3.55 ± 0.20 mm to 3.41 ± 0.20 mm in hyperopic eyes (all *p* < 0.01).

**TABLE 2:** Anterior segment parameters before and after cycloplegia.

		Before cycloplegia	After cycloplegia	*p* value
CCT (µm)	Hyperopia	544.24 ± 34.68	545.00 ± 34.55	0.147^a^
	Emmetropia	534.38 ± 31.54	534.82 ± 30.78	0.548^a^
	Myopia	538.59 ± 33.36	538.98 ± 33.04	0.761^b^
CLR (µm)	Hyperopia	176.97 ± 160.64	32.65 ± 136.28	<0.01^a^
	Emmetropia	3.26 ± 143.39	−35.53 ± 152.72	<0.01^a^
	Myopia	−66.36 ± 151.97	−95.75 ± 143.70	<0.01^b^
CLT (mm)	Hyperopia	3.55 ± 0.20	3.41 ± 0.20	<0.01^a^
	Emmetropia	3.42 ± 0.18	3.38 ± 0.19	<0.01^a^
	Myopia	3.37 ± 0.16	3.34 ± 0.15	<0.01^b^
ARL (mm)	Hyperopia	10.79 ± 1.55	12.81 ± 1.35	<0.01^a^
	Emmetropia	12.13 ± 1.30	12.99 ± 1.41	<0.01^a^
	Myopia	13.02 ± 1.20	13.73 ± 1.14	<0.01^a^
PRL (mm)	Hyperopia	5.67 ± 0.50	5.84 ± 0.45	<0.01^a^
	Emmetropia	5.91 ± 0.45	5.97 ± 0.49	0.160^a^
	Myopia	6.14 ± 0.36	6.07 ± 0.35	0.027^a^
ACD (mm)	Hyperopia	2.97 ± 0.24	3.15 ± 0.23	<0.01^a^
	Emmetropia	3.21 ± 0.19	3.30 ± 0.19	<0.01^a^
	Myopia	3.34 ± 0.20	3.40 ± 0.20	<0.01^b^
ACW (mm)	Hyperopia	11.67 ± 0.40	11.62 ± 0.41	0.318^a^
	Emmetropia	11.65 ± 0.46	11.69 ± 0.45	0.270^b^
	Myopia	11.77 ± 0.48	11.76 ± 0.55	0.891^b^
LD (mm)	Hyperopia	0.23 ± 0.08	0.24 ± 0.09	0.498^a^
	Emmetropia	0.20 ± 0.09	0.21 ± 0.08	0.107^b^
	Myopia	0.25 ± 0.09	0.25 ± 0.11	0.265^b^
LT (°)	Hyperopia	5.38 ± 1.16	5.35 ± 1.10	0.821^a^
	Emmetropia	4.66 ± 1.21	4.82 ± 1.19	0.186^b^
	Myopia	4.11 ± 1.35	4.34 ± 1.55	0.273^b^

a Paired t test.

b Wilcoxon signed-rank test.

CCT, central corneal thickness; CLR, crystalline lens rise; CLT, crystalline lens thickness; ARL, anterior radius of lens; PRL, posterior radius of lens; ACD, anterior chamber depth; ACW, anterior chamber width; LD, lens decentration; LT, lens tilt.

ARL significantly increased from 12.13 ± 1.30 mm to 12.99 ± 1.41 mm in emmetropic eyes, from 13.02 ± 1.20 mm to 13.73 ± 1.14 mm in myopic eyes, and from 10.79 ± 1.55 mm to 12.81 ± 1.35 mm in hyperopic eyes (all *p* < 0.01). ACD significantly increased from 3.21 ± 0.19 mm to 3.30 ± 0.19 mm in emmetropic eyes, from 3.34 ± 0.20 mm to 3.40 ± 0.20 mm in myopic eyes, and from 2.97 ± 0.24 mm to 3.15 ± 0.23 mm in hyperopic eyes (all *p* < 0.01).

PRL significantly increased from 5.67 ± 0.50 mm to 5.84 ± 0.45 mm in hyperopic eyes (*p* < 0.01), although no changes were found in emmetropic eyes and myopic eyes. The CCT, ACW, LD, and LT parameters did not change after cycloplegia in all three refractive groups (all *p* > 0.05).

### Differences in Changes in Anterior Segment Parameters After Cycloplegia Among Emmetropic, Myopic, and Hyperopic Eyes

Changes in anterior segment parameters after cycloplegia among emmetropic, myopic, and hyperopic eyes were compared further (Table 3). Changes in CLR, CLT, ARL, PRL, and ACD in hyperopic eyes were significantly greater than those in emmetropic eyes and in myopic eyes (all *p* < 0.05). In addition, the emmetropic eyes and myopic eyes had similar changes after cycloplegia among all anterior segment parameters, except for that of PRL (*p* < 0.05). No difference in changes of CCT, ACW, LD, and LT among the three refractive groups was found (all *p* > 0.05).

**TABLE 3:** Comparison of changes in anterior segment parameters before and after cycloplegia among emmetropic, myopic and hyperopic eyes.

	Emmetropia (*n* = 34)	Myopia (*n* = 57)	Hyperopia (*n* = 34)	*p* value
CCT (µm)	0.44 ± 4.24	0.39 ± 4.75	0.76 ± 3.00	0.762^a^
CLR (µm)	−38.79 ± 53.28	−29.39 ± 72.76	−144.32 ± 108.85^†‡^	<0.01^a^
CLT (mm)	−0.03 ± 0.03	−0.02 ± 0.03	−0.14 ± 0.10^†‡^	<0.01^a^
ARL (mm)	0.86 ± 0.50	0.71 ± 0.45	2.02 ± 1.32^†‡^	<0.01^a^
PRL (mm)	0.05 ± 0.21	−0.07 ± 0.23^†^	0.18 ± 0.28^†‡^	<0.01^a^
ACD (mm)	0.08 ± 0.04	0.06 ± 0.07	0.18 ± 0.08^†‡^	<0.01^b^
ACW (mm)	0.05 ± 0.28	−0.01 ± 0.27	−0.05 ± 0.28	0.401^b^
LD (mm)	0.01 ± 0.04	0 ± 0.07	0.01 ± 0.06	0.185^b^
LT (°)	0.16 ± 0.90	0.23 ± 1.12	−0.03 ± 0.78	0.654^b^

a One-way ANOVA.

b Kruskal-Wallis test.

^†^ *p* < 0.05 compared to the emmetropia group.

^‡^ *p* < 0.05 compared to the myopia group.

CCT, central corneal thickness; CLR, crystalline lens rise; CLT, crystalline lens thickness; ARL, anterior radius of lens; PRL, posterior radius of lens; ACD, anterior chamber depth; ACW, anterior chamber width; LD, lens decentration; LT, lens tilt.

## Discussion

CASIA2, a newly developed AS-OCT imaging device, enabled us to take detailed biometry measurements in the anterior segment before and after cycloplegia. In this study, we found that CLR, CLT, ARL, and ACD significantly changed after cycloplegia in all three refractive groups. In addition, hyperopic eyes had greater changes in CLR, CLT, ARL, PRL, and ACD after cycloplegia than emmetropic eyes and myopic eyes.

CLR, the perpendicular distance between the line of angle recess and the anterior crystalline lens surface, is an important reference index for the implantation of the pIOL. (Yan et al., 2010) In children, the anterior chamber pIOL and posterior chamber pIOL have been used in the treatment of significant anisometropic myopia and amblyopia. (Pirouzian et al., 2007; Morkos et al., 2021) A CLR of more than 500 µm is correlated with pigment dispersion syndrome after the implantation of anterior chamber pIOL. (Baïkoff, 2006; Yan et al., 2010) In addition, CLR is significantly correlated with central vault distance after ICL surgery. (Gonzalez-Lopez et al., 2019) Short vault distance between the ICL and crystalline lens may induce the formation of contact cataract. (Gimbel et al., 2018) CLT plays a key role in the formation of angle-closure glaucoma. Compared to normal eyes, eyes with angle closure have thicker lenses. (Nongpiur et al., 2011) In this study, we found that the lens moved backward and became thinner after cycloplegia caused by tropicamide in all three refractive groups. Therefore, these changes in CLT and CLR by tropicamide in all refractive statuses should be taken into consideration when determining the suitability for pIOL and ICL surgery and predicting the potential risk of angle-closure glaucoma. Our results were also consistent with those of previous studies. (Yuan et al., 2015; Chen et al., 2020; Mitsukawa et al., 2020) These changes can be attributed to Helmholtz’s theory. (Helmholtz, 1855) During accommodation, as the ciliary contracts, the tension on the zonules is reduced and the lens moves forward. In addition, the mean crystalline lens thickness increases at the same time. (Sheppard et al., 2011) Conversely, as the ciliary muscle is paralyzed, the lens may move backward and become thinner.

ARL and PRL reflect the lens shape and geometry. These parameters can help to understand the mechanism of accommodation of lens. (Pérez-Merino et al., 2015) In this study, ARL increased in all three refractive statuses after cycloplegia, which meant that the anterior surface of the lens became flatter. In addition, PRL increased in hyperopic eyes after cycloplegia, which meant that the posterior surface of the lens became flatter in hyperopic eyes. Our results were consistent with those of previous studies with CASIA2 in which ARL increased and PRL remained unchanged after cycloplegia in myopic subjects. (Chen et al., 2020; Mitsukawa et al., 2020) However, in addition to increasing ARL, we found that PRL also increased after cycloplegia in hyperopic eyes. In accordance to Helmholtz’s theory, the anterior and posterior surface of the lens would become steeper after contraction of the ciliary muscle during accommodation. (Helmholtz, 1855; Du et al., 2012; Pérez-Merino et al., 2015) On the contrary, the anterior and posterior surfaces of the lens may be flatter after cycloplegia.

ACD and ACW are key factors in the diagnosis of glaucoma. (Chen et al., 2013; Loh et al., 2021) For instance, shallow ACD may increase the risk of angle-closure glaucoma. In addition, ACD is a critical parameter in the implantation of pIOL. (Vasavada et al., 2018) Small ACD was significantly correlated with endothelial cell loss after pIOL implantation. (Jonker et al., 2019) In this study, we found that ACD increased in all three refractive groups after cycloplegia. However, no change in ACW was found for each refractive status after cycloplegia. Therefore, changes in ACD by tropicamide should be noted during the evaluation for glaucoma and implantation of pIOL. Our results were also consistent with previous findings. (Chen et al., 2020; Tasci et al., 2021) The increase in ACD after cycloplegia may be attributable to the backward movements of the crystalline lens. A significantly negative relationship between ACD and CLR was identified in various studies. (Chen et al., 2020; Dai et al., 2021)

CCT is a critical factor when making decisions on cornea-based refractive surgery indications. (O'Keefe and Kirwan, 2010) Refractive surgeries such as photorefractive keratectomy (PRK) and laser-assisted *in situ* keratomileusis (LASIK) surgeries were performed in children with refractive amblyopia to enhance stimulation and integration. (Tychsen, 2008; Stahl, 2017; Langenegger et al., 2020) In addition, a thin CCT may indicate the presence of keratoconus. (Yağmur Kanra and Uslu, 2021) In this study, we found that CCT remained unchanged by tropicamide in all refractive statuses. However, the changes in CCT after cycloplegia remain controversial and may vary according to the type of mydriatic and equipments. The 1% cyclopentolate hydrochloride solution may lead to a significant decrease in CCT. (Palamar et al., 2011) Conversely, the effects of 0.5% tropicamide on CCT seem insignificant. (Mitsukawa et al., 2020)

In this study, we found that hyperopic eyes were under greater influence by tropicamide than emmetropic eyes and myopic eyes. Hyperopic eyes showed greater changes in CLR, CLT, ARL, PRL, and ACD. We speculated that these greater changes in hyperopic eyes may be attributed to differences between the cycloplegic and non-cycloplegic spheres in hyperopic eyes and the effects of tropicamide on hyperopic eyes. For one thing, hyperopic eyes had greater differences between cycloplegic and non-cycloplegic spheres than emmetropic eyes and myopic eyes. The difference would also increase with increasing hyperopia. (Doherty et al., 2019; Liu et al., 2021) As anterior segment changes are correlated with SE changes, greater SE changes may lead to greater anterior segment changes. For another thing, tropicamide may have weaker cycloplegic effects on hyperopic eyes compared to other mydriatics. Cyclopentolate, noted for its long-lasting cycloplegic effect, is widely used in clinical practice. (Hashemi et al., 2020) Though the cycloplegic efficacy of 0.5% tropicamide was comparable to that of 1% cyclopentolate in myopic eyes, the cycloplegic efficacy of tropicamide was weaker than cyclopentolate in hyperopic eyes. (Yazdani et al., 2018)

Some limitations remain in our study. First, only children were recruited in our study, but accommodation ability varies according to age, so the effects of tropicamide on different age groups deserve further analysis. Second, the small sample sizes of the three refractive groups should be noted, although there were no significant differences in age or gender among these groups.

In this study, we initially reported on the effects of 0.5% tropicamide on anterior segment parameters with CASIA2 in different refractive statuses. While various anterior segment parameters changed after cycloplegia in all refractive statuses, significant changes in CLR, CLT, ARL, PRL, and ACD in hyperopic eyes after using tropicamide should be noted for proper clinical interpretation in various diseases.

## Data Availability

The original contributions presented in the study are included in the article/Supplementary Material, further inquiries can be directed to the corresponding authors.
